# A review of ovarian-adnexal reporting and data system and radiomics in ovarian cancer: focus on ultrasound applications

**DOI:** 10.3389/fonc.2026.1877133

**Published:** 2026-08-03

**Authors:** Yang Yu, Huidong Li, Ying Liu

**Affiliations:** 1 Department of Radiology, Tianjin Medical University Cancer Institute and Hospital, National Clinical Research Center for Cancer/Tianjin’s Clinical Research Center for Cancer/Key Laboratory of Cancer Prevention and Therapy, Tianjin, China; 2 Department of Ultrasound, Tianjin Central Hospital of Gynecology Obstetrics, Tianjin, China

**Keywords:** imaging diagnosis, O-RADS, ovarian cancer, radiomics, ultrasound

## Abstract

Ovarian cancer (OC) is predominantly diagnosed at advanced stages, highlighting an urgent unmet need for effective early detection. The Ovarian-Adnexal Reporting and Data System (O-RADS) provides standardized criteria for ovarian ultrasound interpretation, and ultrasound based radiomics has evolved into a promising auxiliary modality for OC assessment. Cumulative evidence suggests that integrating radiomics features with O-RADS risk stratification can enhance non-invasive characterization of ovarian tumors, support diagnosis, and help evaluate treatment response. However, several critical barriers hinder its routine clinical implementation, including the lack of multicenter standardization, heterogeneous imaging protocols and poor cross-institutional reproducibility. This review systematically outlines the core diagnostic framework of O-RADS, summarizes recent advances in ultrasound radiomics, and explores the synergistic diagnostic performance of the combined workflow to guide clinical practice and future research endeavors.

## 1 Introduction

With an estimated 225,000 new cases and 145,000 deaths annually, ovarian cancer (OC) persists as one of the most lethal gynecological malignancies globally, contributing to a notably high mortality rate (1). Despite advances in clinical management, the five-year overall survival (OS) rate remains suboptimal. This is highlighted by recent statistics from China which indicated an OS rate of only about 40% (2). Due to the nonspecific clinical symptoms of OC, most of patients are diagnosed at advanced stages, which constitutes the main reason for its high mortality. Therefore, early detection and timely intervention are of critical clinical significance. They enable more complete surgical removal of the tumor and increase sensitivity to platinum-based chemotherapy, which are key to improve treatment outcomes and long-survival.

Ultrasound (US), especially transvaginal US, serves as the first-line imaging modality for the assessment and characterization of adnexal lesions, owing to its wide availability, cost-effectiveness, and real-time evaluation (3). However, achieving accurate differential diagnosis of OC continues to present considerable challenges due to variations in imaging manifestations (4). Moreover, conventional US interpretation is intrinsically subjective and highly reliant on operators experience and expertise, resulting in variable diagnostic accuracy and interobserver discrepancies (5). Against this backdrop, the application of diverse medical imaging diagnostic models for OC screening has garnered increasing attention as a potential strategy to address these limitations.

The Ovarian-Adnexal Reporting and Data System (O-RADS) was created to fulfill the need for a uniform lexicon and a dedicated risk stratification framework specifically tailored for adnexal lesions. Proposed by the American College of Radiology, O-RADS provides a structured reporting system that categorizes lesions according to their sonographic morphological phenotypes and corresponding malignant risk probabilities (6). Equipped with standardized imaging descriptors and matched malignancy risk scoring criteria, O-RADS enhances the diagnostic performance for ovarian adnexal lesions, and significantly improves inter-observer agreement even among radiologists with limited clinical experience in gynecological ultrasonography. As such, O-RADS constitutes a pivotal advancement toward the standardization of evidence-based imaging evaluation for ovarian and adnexal masses. By establishing a consistent framework, it facilitates more efficient workflows, clearer reporting, and ultimately supports earlier diagnosis and individualized patient management.

As an emerging subdiscipline in medical imaging, radiomics extracts high-throughput and high-dimensional quantitative features from conventional imaging (7, 8). It enables the characterization of intratumoral heterogeneity and subtle microscopic structural changes beyond the resolution of human visual perception, offering innovative quantitative strategy for tumor evaluation (9). Integrating radiomic models with the O-RADS framework and clinical data allows for better discrimination of ovarian masses and more precise risk stratification, thereby improving overall diagnostic efficacy (10, 11). Synergizing these models with deep learning also shows promise for automated lesion classification and reduced operator variability (12). This multimodal approach leverages the strengths of US while compensating for its limitations, offering a comprehensive, non-invasive diagnostic tool.

In this review, we systematically elaborate on the conceptual frameworks of O-RADS and ultrasound radiomics, followed by a critical evaluation of their respective technical advances in the clinical management of OC. We further elucidated the unresolved challenges hindering their clinical translation, and put forward standardized analytical pipelines to facilitate subsequent validation research and promote clinical implementation.

## 2 O-RADS application for ovarian cancer ultrasound diagnosis

### 2.1 Classification system and scoring criteria

O-RADS classifies each lesion using a categorical risk score to quantify its malignant potential, which is stratified into three distinct categories: benign (O-RADS 1–2), intermediate-risk (O-RADS 3), and high-risk (O-RADS 4-5) (13). This uniform scoring system minimizes inter-observer variability in lesion interpretation with clear risk-category criteria, thus boosting diagnostic reproducibility significantly (14). From a clinical perspective, O-RADS risk stratification guides evidence-based management of ovarian lesions: low-risk lesions are managed with conservative follow-up; intermediate-risk ones require further diagnostic evaluation or intensified surveillance; and high-risk lesions demand surgical intervention or referral to an oncology specialist. By integrating morphological and vascular features into a composite score, O-RADS facilitates consistent radiologic communication and ultrasound- driven decision-making (15).

### 2.2 Clinical validation and diagnostic performance

O-RADS showed significantly higher sensitivity for malignant adnexal lesions relative to GI-RADS (p=0.003) and IOTA (p=0.0007). The three tools achieved comparable overall inter-observer concordance (κ=0.77 for O-RADS versus 0.69 and 0.63 for GI-RADS and IOTA, respectively), with O-RADS exhibited a trend of higher inter-observer reliability (16). Multiple investigations have validated the diagnostic utility of O-RADS in differentiating benign from malignant ovarian tumors (3, 17). Notably, the diagnostic performance of O-RADS can be further enhanced by incorporating additional indicators, such as contrast-enhanced imaging and serum tumor biomarkers (18–21). However, validation across various studies has revealed certain limitations in the diagnostic performance of the O-RADS classification system. A key constraint is the system’s performance in specific, challenging scenarios. While O-RADS exhibits good diagnostic accuracy for cystic and typical benign lesions, its application can be problematic in distinguishing certain benign solid lesions from malignant ones. Additionally, a significant limitation lies in the substantial overlap observed in color Doppler flow scores between benign and malignant lesions, particularly within Grades 1 and 2 lesions (6, 22). This overlap can limit the system’s specificity in clinical practice, especially in complex or borderline cases where vascular patterns appear ambiguous. Advances in artificial intelligence (AI) present a promising pathway to enhance O-RADS based assessments, as integrating AI with O-RADS could help address these diagnostic challenges.

## 3 Research progress of radiomics in ultrasound imaging of ovarian cancer

### 3.1 Overview

Since its initial proposal by Lambin et al. in 2012, radiomics has emerged as a rapidly evolving interdisciplinary field within medical imaging (23). It exhibits notable merits in both the diagnostic workup of OC and the assessment of treatment response. Compared with computed tomography (CT), magnetic resonance imaging (MRI), or Positron Emission Tomography-Computed Tomography (PET-CT), US-based radiomics encounters unique and more pronounced limitations such as inherent image noise, lower spatial resolution, and a higher degree of variability due to operator dependence and acquisition settings (24, 25). Nonetheless, existing studies have confirmed the promising diagnostic performance of ultrasound-based radiomics in several clinical applications related to ovarian tumors (10, 11).

### 3.2 Applications of ultrasound-based radiomics in ovarian tumors

#### 3.2.1 Differentiation of benign and malignant ovarian tumors

With the advancement of radiomics, it has increasingly demonstrated advantages in the benign and malignant discrimination of adnexal masses. In 2014, the GyneScan system combined 3D ultrasound texture features with traditional machine learning, achieving favorable diagnostic accuracy, and laying the technical foundation for radiomic quantification (26). The AROMA pilot study recruited a total of 241 women with available ultrasonographic images of ovarian masses, among whom the masses were divided into three homogeneous groups: solid, cystic and motley. The TRACE4 radiomic platform enabled a fully automated radiomic analysis pipeline that complied with the Image Biomarker Standardization Initiative (IBSI) guidelines. The constructed predictive models demonstrated robust performance across all lesion subtypes: solid masses yielded an accuracy of 80%, sensitivity of 78%, specificity of 83%, and an AUC of 0.87; cystic reached 87% accuracy, 75% sensitivity, 90% specificity, with an AUC of 0.88; mixed masses attained 81% across accuracy, sensitivity, and specificity, alongside an AUC of 0.89 (27). This method can facilitate the objective identification of OC, complementing or operating independently of the subjective assessment by an ultrasound expert.

Unlike simple binary classification of benign and malignant tumors, ovarian tumors pose considerable challenges in both clinical and pathological diagnosis because of their diverse histological origins and complex biological behaviors. The existence of borderline tumors (also referred to as tumors of low malignant potential) further highlights the unique complexity involved in diagnosing and treatment of this disease. A prior study confirmed that ultrasound-based radiomic models effectively distinguish benign from borderline/malignant serous ovarian tumors and borderline from malignant ones, surpassing senior sonographers (28). It is worth noting that although the study categorized ovarian tumors into three types (benign, malignant, and border-line) based on pathology, the final analysis still treated them as a dichotomous variable. In another study, Du et al. developed an US-based multiclass prediction radiomic signature (Rad_Sig) to differentiate benign, borderline, and malignant ovarian tumors. Unfortunately, the Rad_Sig three-class prediction model failed to accurately identify borderline ovarian tumors, and the precision, recall, and F1 scores for this class were all zero (29).

#### 3.2.2 Histological classification and gene mutation

Integrating US-based radiomic features with clinical indicators allows for effective preoperative differentiation between type I and type II epithelial ovarian cancer, and the predictive performance of the combined models (AUC: 0.82-0.98) is significantly better than that of models using either radiomic or clinical data alone (30–32). Yang et al. further constructed and validated ultrasound radiomic classifiers to distinguish specific subtypes of OC. The results demonstrated that while US-based radiomic models demonstrated limited efficacy in distinguishing clear cell carcinoma, endometrioid carcinoma, and low-grade serous carcinoma, they performed well in identifying high-grade serous carcinoma (HGSC) and mucinous carcinoma, with HGSC predictions showing high clinical consistency and net benefit (33). Recently, a multi-task preoperative OC prediction platform termed OVUCM was established via multimodal fusion of ultrasound, contrast-enhanced CT, MRI and clinical information (34). This unified model produced robust AUCs ranging from 0.847 to 0.929 across five stratified diagnostic tasks: benign versus non-benign lesions, borderline versus malignant tumors, non-epithelial versus epithelial subtypes, FIGO stage I–II versus III–IV disease, and non-HGSOC versus HGSOC. Clinically, OVUCM attained a significantly higher AUC than institutional multidisciplinary team (MDT) consensus for non-HGSOC/HGSOC) differentiation (p < 0.05). For histological subtypes discrimination (non-epithelial vs. epithelial and non-HGSOC vs. HGSOC), the system outperformed all clinicians (all p < 0.05). Thus, multimodal radiomics substantially improve the preoperative histological subtyping and stratification of ovarian tumors, outperforming single-modality radiomic tools and routine clinical evaluation by individual clinicians or MDTs.

US-based radiogenomics, defined as the correlation of ultrasound imaging features with genomic data, is a promising and increasingly investigated field (35). Nero et al. proposed a new screening strategy that employs a machine learning model applied to ultrasound images for the preselection of women eligible for BRCA genetic testing. More specifically, with a specificity of 0.87, the described model as a large-scale screening tool would yield 13 false positives per 100 gBRCA1/2 wild-type patients, thereby referring only this small subset to unnecessary genetic screening. It facilitates the identification of eligible candidates for clinical genetic testing, thus effectively optimizing the testing process and resource allocation (36). A subsequent cost-effectiveness analysis found that combining the model with clinical criteria could significantly lower BRCA-related cancer incidence more cost-effectively than current methods, supporting its future integration into prevention protocols (37). Given that mutations in the BRCA pathway are linked to divergent treatment outcomes in epithelial ovarian cancer, this model provides a basis for a novel approach to tumor assessment, though its predictive power requires further optimization.

#### 3.2.3 Metastatic risk and prediction of treatment response

The diagnostic utility of ultrasound in evaluating OC metastatic risk has been proved by studies, with its diagnostic performance comparable to that of CT. However, its sensitivity remains low (38, 39). The development of radiomics has provided a novel solution to this problem. Qi et al. investigated the feasibility of predicting lymph node metastasis in patients with high grade serous OC by integrating ultrasound-based radiomics with preoperative clinical characteristics. The model achieved an AUC of 0.930 in the training cohort and 0.881 in the external test cohort, with corresponding sensitivities of 0.83 and 0.71 respectively (40). In addition to its application in evaluating lymph node metastasis, ultrasound-based radiomics also demonstrates advantages in assessing peritoneal metastasis. In a multicenter retrospective study of 619 OC patients, a radiomic nomogram integrating transvaginal ultrasound radiomic and multimodal clinical data demonstrated strong performance in diagnosing peritoneal metastasis. The model achieved AUC values of 0.912, 0.883, and 0.831 in the training, internal test, and external test sets, respectively (41). The nomogram showed significantly better diagnostic performance compared to single modality models. This multi-modal approach supports a comprehensive and objective assessment of OC staging prior to surgery. This enhanced preoperative insight facilitates the formulation of personalized therapeutic strategies, including optimized surgical planning and selection of neoadjuvant chemotherapy, thereby advancing the management of OC toward greater precision and personalization.

US-based radiomics has also showed prominent advantages in predicting treatment outcomes, which further enhances the foundation for personalized treatment strategies. For instance, Li et al. developed a predictive model combining ultrasound-based radiomic features, laboratory biomarkers, and clinical variables to assess treatment response in patients with advanced epithelial OC. This integrated model outperformed counterparts built exclusively on clinical data or radiomis features alone, attaining reliable performance metrics in the external validation set with an AUC of 0.82, accuracy of 0.84, sensitivity of 0.80, specificity of 0.75, precision of 0.88, positive predictive value (PPV) of 0.81, negative predictive value (NPV) of 0.86, and an F1 score of 0.78 (42). Tumor therapeutic strategies are closely intertwined with the tumor microenvironment, as metabolic reprogramming represents the fundamental driver of tumor invasion, malignant progression, and the development of therapeutic resistance. Ultrasound radiomics designed for therapeutic response prediction may facilitate noninvasive tumor microenvironment characterization. Exploring the associations between ultrasound radiomic phenotypes and specific metabolic pathway alterations can deliver biologically meaningful evidence for noninvasive risk stratification, thereby connecting quantitative imaging with underlying biological characteristics of malignancy (43).

Nevertheless, it should be acknowledged that current research on ovarian metastasis and treatment response has predominantly focused on epithelial OC, which fails to encompass the full spectrum of ovarian malignancies. As previously highlighted, the underexplored utility of ultrasound radiomics in non-epithelial ovarian tumor subtypes remain largely unverified, constituting a critical research gap in existing studies. Non-epithelial ovarian malignancies exhibit distinct sonographic features compared with epithelial subtypes; these rare tumors typically present as solid masses with well-circumscribed margins and heterogeneous echotexture, which is markedly different from the cystic-solid or cyst-dominant morphology patterns that characterize most epithelial ovarian cancers (44). Such inherent inter-subtype discrepancies in ultrasound imaging phenotypes, accompanied by phenotypic overlap between different ovarian tumor types, introduce systematic biases in radiomic feature extraction. Variations in tissue composition alter the quantitative distribution of ultrasound texture and first-order features across histological subgroups, substantially diminishing the subtype-specific discriminability of ultrasound radiomic signatures and impairing the cross-subtype generalizability of diagnostic models primarily trained on epithelial OC datasets.

## 4 The integration of O-RADS and ultrasound radiomics

O-RADS provides clinicians with a standardized diagnostic framework to unify evaluation criteria, while ultrasound-based radiomics complements this grading system with objective quantitative imaging biomarkers. Their complementary strengths lay a solid foundation for comprehensive evaluation of ovarian cancer malignancy and metastatic potential. To integrate these two valuable modalities for optimized predictive performance, researchers have proposed three mainstream multimodal fusion pipelines for joint modeling, starting with two-stage hierarchical architectures that first stratify lesions by O-RADS categories before feeding ultrasound images into radiomic models for refined risk stratification. This approach reduces computational burden and fits routine clinical diagnostic workflows. For instance, Xie et al. constructed a deep learning model specifically for O-RADS 4 lesions to distinguish benign and malignant adnexal masses, with multicenter validation confirming reliable discriminatory power that effectively reducing unnecessary surgical interventions for patients with benign intermediate-risk lesions (45). A second widely adopted paradigm corresponds to end-to-end integrated deep learning frameworks, which encode categorical O-RADS grades as covariates and concatenate these graded labels with latent ultrasound deep imaging features within neural network hidden layers to jointly train a unified ensemble classifier. One relevant study integrated O-RADS scores with deep radiomic signatures, achieving an AUC of around 0.97 in both internal and external validation cohorts (12). However, this method carries a potential drawback that the strong predictive weight of subjective O-RADS grading may mask inherent imaging features, making the model outputs overly reliant on human visual assessment. The third fusion strategy operates at the feature level, where discretized O-RADS grades are transformed into one-dimensional feature vectors and combined with high-dimensional ultrasound radiomic profiles. The merged feature matrix undergoes dimensionality reduction via algorithms such as LASSO regression prior to classifier input. This fusion strategy can retain the independent predictive value of both clinical grading and imaging-derived predictors. A previous study screened valuable O-RADS and radiomic predictors using LASSO regression to establish a combined nomogram, whose AUC was significantly superior to that of single-modality prediction models (11).

With the assistance of deep learning, a comprehensive model that integrates clinical factors (age, menstrual status, tumor diameter, and CA-125, O-RADS scores, and deep learning radiomic model predictions demonstrated superior diagnostic performance for OC compared to standalone clinical models and clinical O-RADS models. This improvement was confirmed by net reclassification improvement (NRI) and integrated discrimination improvement (IDI) metrics. Furthermore, the ensemble model maintained favorable diagnostic accuracy in both subgroups, including the normal CA-125 cohort and the cohort with tumor diameter <10 cm, highlighting its considerable clinical potential for the preoperative diagnosis of OC (12). The implementation of this model also substantially enhanced the diagnostic precision and reliability of sonographers. The average AUC of sonographers improved by 11% in the internal validation set and by 7.7% in the external validation set. This paves the way for the early diagnosis of OC and elevates the diagnostic performance of ultrasonographers. Additionally, progress in automatic segmentation technology further facilitates the objective stratification of ORADS, reducing the time spent on image segmentation and minimizing subjectivity, thereby further optimizing the radiomic model for OC diagnosis (46, 47).

Accumulating evidence reveals that multimodal imaging fusion alone fails to fully satisfy comprehensive clinical demands for ovarian lesion diagnosis, therapeutic stratification, and long-term prognostic evaluation. To address such limitations, Zhang et al. proposed a three-tier interdisciplinary assessment system encompassing molecular biomarkers, functional readouts, and multimodal imaging signatures, which provides important translational references for oncological imaging research (48). Such cross-disciplinary multidimensional analytical frameworks enable comprehensive characterization of tumor heterogeneity. Similarly, the combination of standardized O-RADS structured reporting and radiomics-derived quantitative imaging features represents another parallel approach to capture heterogeneous tumor phenotypes, refine personalized risk stratification, and streamline clinical decision-making throughout the full workflow of OC management.

## 5 Technical challenges and limitations

The integration of O-RADS and ultrasound-based radiomics holds notable diagnostic utility in OC, yet existing relevant investigations are hampered by multiple modality-specific and translational limitations. Foremost, ultrasound acquisition parameters including transducer frequency, gain adjustments and focal positioning directly reshape lesion speckle patterns and grayscale distribution, triggering divergent texture feature values and severely compromising cross-device feature reproducibility. Beyond variable scanning settings, ultrasound imaging inherently generates speckle noise, which distorts texture quantification and destabilizes extracted radiomic signatures; inter-scanner hardware discrepancies, site-specific scanning protocols and inter-operator technical variability further compound speckle heterogeneity and degrade overall image consistency. Operative subjectivity constitutes another core ultrasound-specific confounder: inconsistent transducer compression, arbitrary scanning plane selection, respiratory and peristaltic motion artifacts shift the spatial arrangement of tissue scatterers and echo phases, distorting speckle appearances and amplifying noise-driven feature fluctuations, while inter-sonographer discrepancies during ROI segmentation additionally weaken measurement consistency across datasets.

Apart from these modality-specific technical confounders, two broader translational bottlenecks persist. Regulatory and privacy limits hinder multicenter data sharing and access to diverse training cohorts. Limited interpretability remains a barrier to wider clinical adoption of radiomics, as physicians tend to hesitate when incorporating models with opaque decision workflows that lack transparent validation.

Therefore, prospective and multicenter validation studies are urgently needed to verify the clinical value of O-RADS combined ultrasound radiomics and guarantee robust reproducibility, thereby establishing solid evidence to support clinical translation. Consistently, emerging research focusing on standardized quantitative imaging modeling underscores the necessity of unified scanning protocols and rigorous external validation to stabilize computational biomarkers, and such standardized methodological frameworks are directly transferable to the construction of ultrasound-based radiomic signatures (49).

## 6 Conclusion

In the field of imaging diagnosis and clinical management of ovarian lesions, both the O-RADS and radiomics offer significant and complementary value. O-RADS improves ultrasound report consistency and facilitates inter-institutional communication via standardized terminology, risk stratification, and clear management guidelines, solidifying its role as an essential tool in ovarian tumor imaging evaluation. Radiomics complements and advances traditional visual assessment by extracting deep feature information on tumor heterogeneity, texture, and morphology. Future developments should prioritize several key areas. First, radiomic features should be leveraged to refine O-RADS risk stratification and to provide finer subclassification of lesions, thereby enabling the development of more robust integrated diagnostic models. Next, these advances must be translated into clinical practice via user friendly and interpretable decision support systems to ensure seamless adoption into diagnostic workflows. Finally, combining radiomics with multimodal imaging, such as ultrasound elastography and functional MRI, and integrating it with multiomics data (e.g. genomics, pathomics) will be critical for building a more comprehensive framework for disease evaluation and management.
